# Temporal Trends in Cardiovascular Health Metrics in Italy, 2015–2024: A Ten-Year Report from the Longevity Check-Up (Lookup) 8+ Study

**DOI:** 10.3390/medsci13040251

**Published:** 2025-10-30

**Authors:** Stefano Cacciatore, Elena Levati, Riccardo Calvani, Matteo Tosato, Francesca Ciciarello, Vincenzo Galluzzo, Sara Salini, Andrea Russo, Emanuele Marzetti, Francesco Landi

**Affiliations:** 1 Department of Geriatrics, Orthopedics and Rheumatology, Università Cattolica del Sacro Cuore, Largo Francesco Vito 1, 00168 Rome, Italy; elena.levati1@guest.policlinicogemelli.it (E.L.); riccardo.calvani@unicatt.it (R.C.); francesco.landi@unicatt.it (F.L.); 2 Fondazione Policlinico Universitario “Agostino Gemelli” IRCCS, Largo Agostino Gemelli 8, 00168 Rome, Italy; matteo.tosato@policlinicogemelli.it (M.T.); francesca.ciciarello@policlinicogemelli.it (F.C.); vincenzo.galluzzo@policlinicogemelli.it (V.G.); sara.salini@policlinicogemelli.it (S.S.); andrea.russo1@policlinicogemelli.it (A.R.)

**Keywords:** cardiovascular health, Life’s Simple 7, health behavior, risk factors, COVID-19 pandemic, health surveys, primary prevention, public health surveillance

## Abstract

Background/Objectives: The objective of this ten-year report is to describe temporal trends in the cardiovascular health (CVH) score and its individual components across ages and sexes. We also examined the impact of the post-COVID-19 period on ideal CVH and identified demographic predictors of favorable cardiovascular risk profiles. Methods: Data for this cross-sectional study were collected between 2015 and 2024 as part of the Lookup 8+ project, an ongoing initiative integrating field-based CVH assessments across Italy. CVH was operationalized using a modified CVH score (0–7 points) inspired by Life’s Simple 7, combining behavioral and clinical metrics. Trends over time and across demographic groups were examined using descriptive statistics and multivariable models adjusted for age, sex, and year of assessment. Results: The study included 18,491 participants (mean age 56.1 ± 14.8 years; 55.2% women). After an initial decline in CVH score between 2015 and 2017 (mean score from 4.39 to 3.95), a gradual improvement followed, reaching 4.41 in 2024. Younger adults (18–39 years; 71.9% in 2024) and women (56.8%) consistently showed the highest prevalence of ideal CVH (score ≥ 5). The post-COVID-19 period was independently associated with higher odds of ideal CVH (OR 1.32; 95% CI 1.24–1.40). While blood pressure and cholesterol metrics improved, dietary quality and glycemic control worsened over time. Conclusions: From 2015 to 2024, overall CVH improved among Lookup participants, particularly among younger individuals after the COVID-19 pandemic. However, substantial age- and sex-related gaps remain, requiring targeted and equity-oriented prevention efforts.

## 1. Introduction

Cardiovascular disease (CVD) is the leading cause of mortality and disability worldwide, accounting for approximately one-third of global deaths and contributing to 34.4 million years lived with disability in 2019 [1]. This burden is primarily driven by modifiable risk factors that accumulate throughout the life course, including high systolic blood pressure, elevated non-HDL cholesterol, excess body weight, diabetes, physical inactivity, unhealthy dietary patterns, and tobacco use [2]. To address this burden, the American Heart Association introduced the concept of ideal cardiovascular health (CVH), first via Life’s Simple 7 (LS7) [3], later revised into Life’s Essential 8 (LE8) [4]. These frameworks integrate behavioral and clinical factors and provide a standardized tool to monitor cardiovascular risk and support prevention [5,6]. In Italy, national surveillance systems such as PASSI (for adults aged 18 to 69 years) and PASSI d’Argento (for those over 70 years of age) have provided valuable data on individual cardiovascular risk factors [7]. However, integrated assessments of overall CVH, based on composite scores such as LS7 or LE8 are not yet widely adopted, especially across the full adult age spectrum and in non-institutional cohorts. Italy has undergone profound sociodemographic shifts over the past decade, including population aging, rising rates of multimorbidity, and the disruptive effects of the COVID-19 pandemic, which may have shaped cardiovascular risk profiles across age groups [8]. Understanding how CVH metrics have evolved in this context is essential to inform prevention strategies and promote CVH in aging societies. The Longevity Check-up Project (Lookup 7+/8+) is an ongoing, large-scale observational study initiated in Italy in June 2015. Its primary objectives are to assess CVH in the adult population and monitor its distribution and association with physical performance and body composition [9].

The objective of this ten-year report is to describe temporal trends in the CVH score and its individual components across three age groups (18–39, 40–64, and ≥65 years) and by sex. We further assessed the impact of the post-COVID-19 period on ideal CVH and identified demographic predictors of favorable cardiovascular risk profiles.

## 2. Materials and Methods

### 2.1. Study Design and Sample Selection

This retrospective cross-sectional analysis is based on data from the Lookup project, a nationwide observational initiative coordinated by the Università Cattolica del Sacro Cuore and Fondazione Policlinico Universitario “Agostino Gemelli” IRCCS (Rome, Italy). We included adults (≥18 years) assessed for CVH metrics from 1 June 2015 to 1 November 2024. Participants were recruited through walk-in campaigns conducted in public spaces (e.g., city squares, malls, cultural events) across various Italian regions. In larger metropolitan areas (population > 250,000), recruitment events were held in multiple neighborhoods to maximize demographic diversity. Individuals were excluded if they self-reported pregnancy, declined capillary blood testing for cholesterol and glucose, or failed to provide written informed consent. All procedures were approved by the Ethics Committee of the Università Cattolica del Sacro Cuore (Protocol #A.1220/CE/2011, approval date: 3 March 2014), and all participants signed informed consent prior to inclusion. Detailed information on the study rationale and methodology has been reported elsewhere [9,10,11].

### 2.2. Data Collection

Lifestyle habits were recorded through a structured interview. Smoking status was categorized as current, former, or never smoker. Body weight was measured using an analog scale, while height was assessed with a stadiometer; body mass index (BMI) was calculated as weight (kg) divided by height squared (m^2^). Dietary intake was evaluated through a simplified food frequency questionnaire addressing major food groups [12], with portion sizes estimated according to Italian standard references [13]. Physical activity was defined as engaging in any form of exercise for ≥30 min, ≥2 times/week in the previous year. This definition, although less stringent than current World Health Organization’s (WHO) recommendations, was chosen to enable rapid and standardized assessment across diverse settings [14]. Information on activity type was also collected. For analytical purposes, participants were classified as physically active or inactive. Capillary blood was drawn via fingertip puncture using sterile, single-use devices. Participants were asked whether they had fasted for at least two hours to distinguish fasting from non-fasting measurements. Total cholesterol and blood glucose were measured using portable point-of-care devices with disposable strips (MultiCare-In, Biomedical Systems International srl, Florence, Italy). Blood pressure was assessed in a seated position using an automated sphygmomanometer.

### 2.3. Definition of Ideal Cardiovascular Health Metrics

Ideal CVH was defined according to an adapted version of the LS7 framework [3,15]. Each of the seven components was dichotomized as ideal (1 point) or non-ideal (0 points), generating a composite CVH score ranging from 0 to 7. Participants were classified as non-smokers if they reported either never smoking or having quit smoking at least 12 months prior to the assessment. A BMI between 18.5 (21.0 for those ≥70 years) and 24.9 kg/m^2^ was used to define normal weight. The higher cutoff for older participants (BMI ≥ 21.0 kg/m^2^) was selected based on prior literature on older adults indicating that lower BMI values are associated with increased mortality and reflect age-related changes in body composition [16]. Participants with BMI values below the selected cutoffs were classified as having non-ideal BMI within the CVH score. Untreated blood pressure values < 120/80 mmHg were considered indicative of optimal blood pressure status. Similarly, total cholesterol levels < 200 mg/dL and glucose < 100 mg/dL when fasting, or <200 mg/dL when non-fasting, in the absence of lipid- or glucose-lowering medications, were classified as ideal. Engaging in any form of exercise lasting at least 30 min on two or more days per week during the previous year was considered ideal physical activity. Dietary habits were classified as ideal in individuals reporting the consumption of at least 3 to 4 servings of fruits and vegetables per day. While this criterion does not fully align with WHO dietary recommendations [17], it served as a pragmatic proxy for healthy eating within the constraints of brief, self-reported evaluation. Based on the total score, participants were categorized into three CVH groups: low (0–2), intermediate (3–4), and high (5–7). Only participants with complete data across all seven CVH metrics were retained for the analysis, following a complete-case approach without imputation.

### 2.4. Statistical Analysis

Descriptive statistics were used to summarize characteristics of the study population, reported overall and stratified by age group. Continuous variables were expressed as mean ± standard deviation, and categorical variables as absolute counts and percentages. Temporal trends in CVH were explored using two complementary approaches. The standardized CVH score was computed annually from 2015 to 2024 and presented as a continuous variable. Annual mean values were examined overall and stratified by age and sex. In parallel, the score was categorized into low (0–2), intermediate (3–4), and high (5–7) levels, and the proportion of participants within each category was calculated across calendar years. To test the association between year and CVH score, we used multivariable linear regression, adjusting for age and sex. To evaluate the determinants of ideal CVH (score ≥ 5), we conducted multivariable logistic regression analyses including age, sex, and a dichotomous indicator for the post-COVID-19 period (defined as calendar year ≥ 2020). Interaction terms were included to test whether the association of the post-COVID-19 period with ideal CVH varied by age or sex. Subsequently, we assessed trends in the prevalence of each of the seven non-ideal CVH metrics from 2015 to 2024, stratified by age group and sex. To support the descriptive findings, we fitted separate logistic regression models for each metric, adjusting for age, sex, and the post-COVID-19 period. Finally, to synthesize differences across metrics, we calculated the absolute difference in prevalence between 2015 and 2024 for each non-ideal CVH factor, stratified by age and sex. Differences were visualized in a summary heatmap. Statistical significance was set at a *p*-value < 0.05. All analyses were performed using R software, version 4.2.3 (R Core Team, Vienna, Austria).

## 3. Results

**Figure 1:**
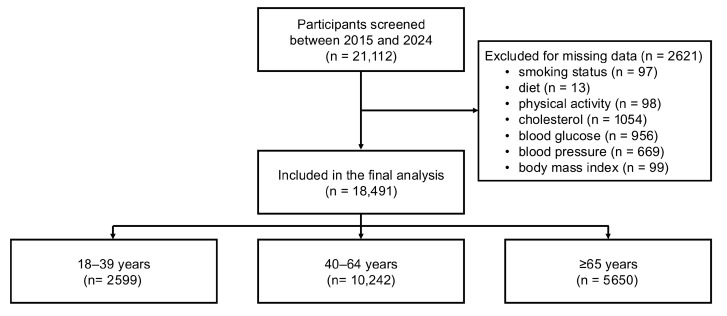
Flowchart illustrating sample selection.

Between 1 June 2015 and 1 November 2024, a total of 21,112 participants aged 18 years or older were enrolled across Italy. Of these, 2621 individuals were excluded due to missing data in one or more variables of interest (Figure 1). The main characteristics of the excluded participants did not differ from those included in the analysis.

The final study sample encompassed 18,491 participants (mean age 56.1 ± 14·8 years; 55.2% women). Participants aged 18–39 years represented 14.7% of the sample (*n* = 2599), those aged 40–64 years 57.2% (*n* = 10,242), and those ≥65 years 31.4% (*n* = 5650). Age-stratified characteristics are reported in Table 1. CVH scores progressively declined according to age, with the prevalence of an ideal score (≥5) halving from 63.9% in younger adults to 29.6% in those 65 years or older. Age-related gradients were observed for BMI, blood pressure, lipid and glucose levels, and the use of blood pressure-, lipid-, and glucose-lowering therapies (Supplementary Table S1).

**Figure 2:**
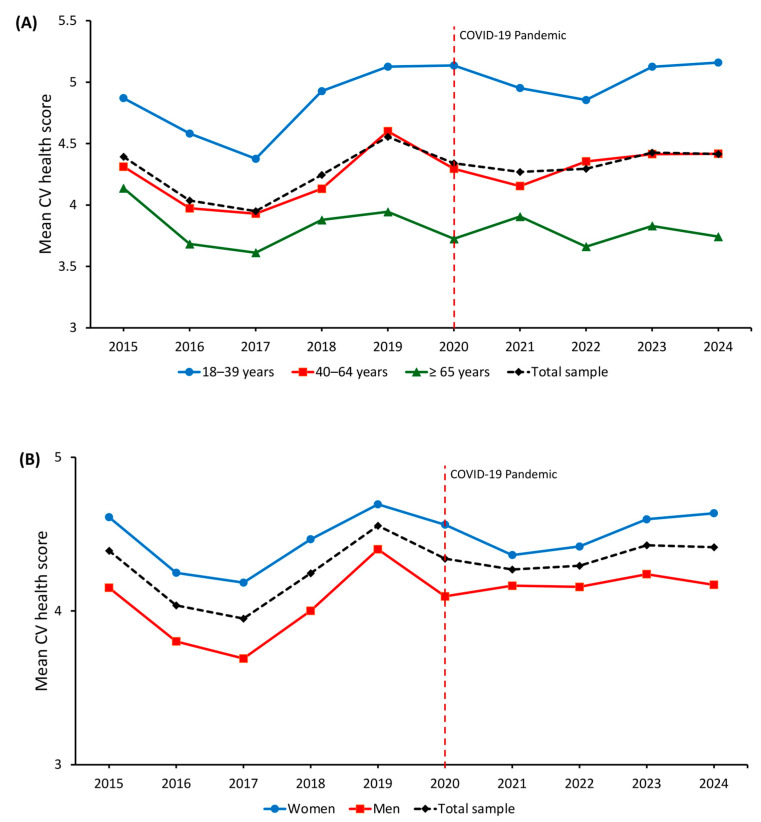
Temporal trends in standardized cardiovascular health score from 2015 to 2024 by (**A**) age and (**B**) sex. The red dashed line indicates the start of the COVID-19 pandemic.

Figure 2 shows age- and sex-standardized CVH score trends from 2015 to 2024. After an initial decline between 2015 and 2017 (mean score from 4.39 to 3.95), a gradual improvement followed, reaching 4.41 in 2024. Younger adults consistently showed higher scores, increasing from 4.87 in 2015 to 5.16 in 2024, while older adults never exceeded a mean of 4.14. When stratified by sex, women consistently showed higher CVH scores than men throughout the study period (4.64 vs. 4.17 in 2024), with a relatively stable difference across calendar years.

Multivariable linear regression models adjusted for age and sex (Supplementary Table S2) confirmed a significant decline in CVH scores in 2016 (β = −0.34; 95% CI −0.43 to −0.26; *p* < 0.001) and 2017 (β = −0.41; 95% CI −0.48 to −0.34; *p* < 0.001) compared with 2015. A modest increase was observed in 2019 (β = 0.16; 95% CI 0.05 to 0.28; *p* = 0.005), while estimates for 2020–2024 were attenuated, suggesting a plateau. Female sex was associated with higher CVH scores (β = 0.40; 95% CI 0.36 to 0.43; *p* < 0.001), whereas older age was associated with a progressive decline (β = −0.65 for age 40–64; β = −1.10 for age ≥ 65, both *p* < 0.001).

**Figure 3:**
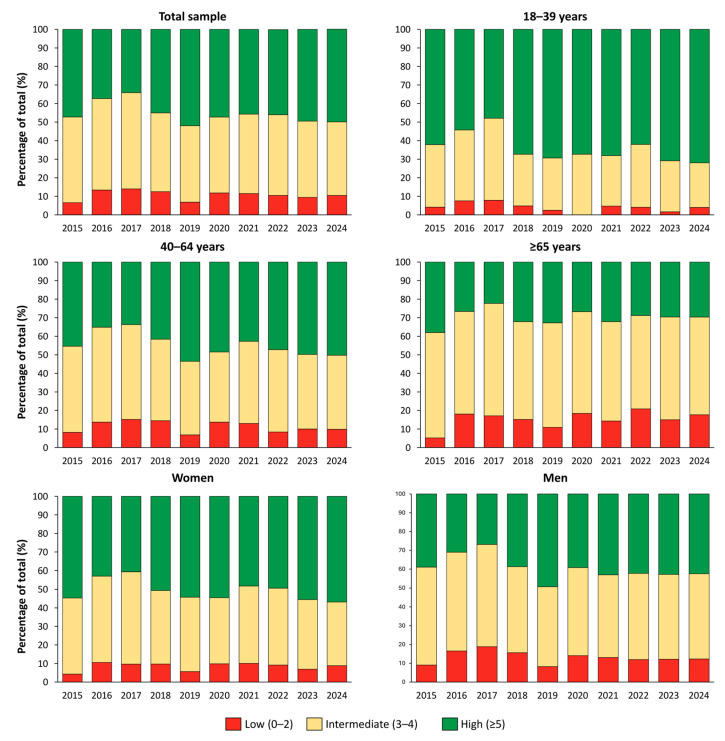
Standardized distribution of cardiovascular health categories in the Italian population from 2015 to 2024.

Figure 3 shows the temporal distribution of CVH scores from 2015 to 2024. The proportion of participants with high CVH (score ≥ 5) decreased from 47.2% in 2015 to 34.1% in 2017, then progressively increased to 50.0% by 2024. The prevalence of low CVH (score 0–2) peaked in 2017 at 14.0% and subsequently declined to 10.5% in 2024. When stratified by age group, younger adults (18–39 years) consistently showed the highest prevalence of high CVH, increasing from 58.3% in 2015 to 71.9% in 2024. Among participants aged 40–64 years, the prevalence declined from 45.3% in 2015 to 33.7% in 2017, followed by a gradual increase to 50.2% in 2024. In adults aged ≥65 years, the proportion of high CVH remained lower throughout the study period, with values ranging from 26.8% in 2020 to 29.7% in 2024, indicating only limited improvement in this subgroup.

Multivariable logistic regression analyses (Table 2) indicated that age, sex, and year of assessment were independently associated with the likelihood of achieving ideal CVH. In Model 1, increasing age was associated with lower odds of ideal CVH (OR 0.97; 95% CI 0.96–0.97; *p* < 0.001), while female sex was associated with higher odds compared to male sex (OR 1.64; 95% CI 1.55–1.75; *p* < 0.001). The post-COVID-19 period was also associated with higher odds of ideal CVH (OR 1.32; 95% CI 1.24–1.40; *p* < 0.001). Model 2 included an interaction term between age and the post-COVID-19 period. The interaction was statistically significant (OR 0.99; 95% CI 0.99–1.00; *p* < 0.001), indicating a slight attenuation of the inverse association between age and ideal CVH in the post-pandemic years. Model 3 tested the interaction between sex and the post-COVID-19 period, which was not statistically significant (OR 0.97; 95% CI 0.86–1.10; *p* = 0.600). In Model 4, the age × post-COVID interaction remained significant, while the sex × post-COVID interaction remained nonsignificant. The associations of age, sex, and year of assessment with ideal CVH did not substantially change across all models, with similar direction and effect sizes.

Temporal trends in the prevalence of individual non-ideal CVH metrics are presented in Supplementary Figures S1–S14. Although year-to-year variability was observed, several consistent patterns emerged across the study period. Active smoking displayed year-to-year fluctuations, with an apparent peak in 2021, particularly among younger adults, followed by a decline in subsequent years. The prevalence of unhealthy diet remained elevated throughout, with higher values among younger participants and men. Physical inactivity first gradually decreased over time, with subsequent modest improvements in women. The proportion of participants with non-ideal total cholesterol and elevated blood pressure declined steadily, especially in women and individuals aged 40–64 years. In contrast, non-ideal glycaemic control increased over the decade, with the sharpest rises observed in older adults and men. A modest decrease in non-ideal BMI was also observed, more evident among younger adults. Supplementary Table S3 reports adjusted logistic regression models evaluating associations of age, sex, and the post-COVID-19 period with each CVH component. Older age was positively associated with non-ideal glycemic control, blood pressure, cholesterol, and BMI, and inversely associated with smoking and poor diet. Female sex was associated with lower odds of non-ideal BMI, blood pressure, and glycemic control, but higher odds of physical inactivity and non-ideal cholesterol. The post-COVID-19 period was associated with lower odds of non-ideal physical activity, elevated blood pressure, non-ideal cholesterol, and BMI. Conversely, higher odds were observed for unhealthy diet and non-ideal glycemic control.

**Figure 4:**
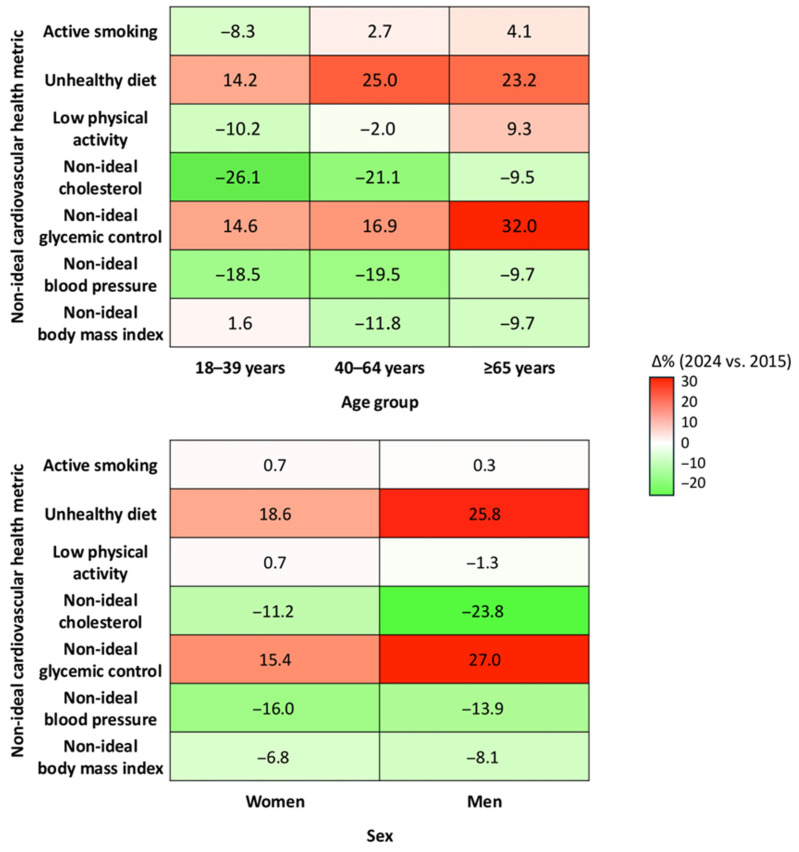
Changes in the prevalence of non-ideal cardiovascular health metrics from 2015 to 2024, stratified by age group and sex. Each cell represents the absolute difference in standardized prevalence (%) of non-ideal status for the corresponding metric between 2024 and 2015.

As shown in Figure 4, improvements occurred in blood pressure and cholesterol values across all age groups, especially among adults aged 40–64 and younger individuals. In contrast, blood glucose values worsened substantially across calendar years, particularly among older adults (+32.0%) and men (+27.0%). Healthy dietary habits also declined across all groups. Changes in smoking, physical activity, and BMI were less consistent, with small improvements among younger adults and minor deteriorations in older persons.

## 4. Discussion

In this large cross-sectional study of over 18,000 adults assessed between 2015 and 2024, we observed significant temporal variation in CVH metrics across age groups and sexes. Following an initial decline, CVH scores gradually improved, with higher values in 2024 compared with the first study year. Nevertheless, disparities by age and sex remained evident, with younger individuals and women consistently showing more favorable profiles.

These findings are consistent with Italian national surveillance data from PASSI and PASSI d’Argento [7], which have documented persistent gaps in CVH by age, sex, and socioeconomic status. While methodological differences exist, particularly regarding measurement approaches, both systems highlight the challenge of promoting ideal CVH metrics at the population level. Compared with earlier estimates, the present study suggests a modest improvement in overall CVH over the past decade, suggesting a gradual shift toward more favorable CVH profiles. Other Italian surveys, including those from Osservatorio Epidemiologico Cardiovascolare, reported high prevalence of major risk factors and similar age and sex gradients [18,19,20,21]. In the United States, fewer than 1% of adults met all seven LS7 metrics between 2003 and 2008 [22]. More recent estimates using LE8 report a mean score of 64.7 out of 100 [23]. Similar trends are evident across Europe and in other global cohorts [5,24]. These findings reflect the persistent gap between CVH targets and population-level indicators.

Comparable evidence from other populations supports our findings. Mean CVH scores, as assessed through LS7 or LE8, remain suboptimal across the United States and Europe, with consistent sex-, age-, and socioeconomic-related disparities. In the United States, the American Heart Association’s LE8 estimates from NHANES 2013–2018 reported a mean adult score of 64.7 out of 100, with women achieving higher scores than men (67.0 vs. 62.5), and diet, physical activity, and BMI identified as the lowest-scoring components [23]. Recent analyses confirm persistent racial and ethnic gaps and minimal post-pandemic improvement in overall CVH, despite favorable trends in smoking and lipid control [25,26,27,28]. In Europe, large population-based cohorts show similar patterns. Data from the UK Biobank study, including 137,794 participants free of cardiovascular disease, demonstrated that higher LE8 scores were strongly associated with lower risks of coronary heart disease, stroke, and overall CVD, with the protective associations being more pronounced in women and younger adults [29]. Longitudinal data from the MONICA project revealed marked reductions in smoking prevalence among men, stable trends among women, and consistent declines in systolic and diastolic blood pressure and total cholesterol between 1985 and 2017, particularly in Central and Eastern Europe, alongside improved hypertension awareness and control [30,31,32]. In contrast, EPIC-based analyses (EPIC-Heart and EPIC-Norfolk) primarily characterized cross-sectional differences by sex and age, showing more favorable profiles in women and younger adults but no consistent longitudinal improvements in overall cardiovascular risk metrics [33,34]. Available evidence from UK cohorts instead indicates stagnation or worsening of key cardiometabolic indicators during and after the pandemic (2020–2022) [35]. Compared with these cohorts, the Lookup 8+ study revealed parallel gradients by age and sex, but slightly higher overall CVH levels, likely reflecting differences in sampling strategy and the inclusion of community-dwelling volunteers rather than clinical populations. Nonetheless, as observed in other countries, our data confirm that population CVH remains far from optimal, underscoring the need for harmonized surveillance systems and cross-country strategies to strengthen cardiovascular prevention.

The observed post-pandemic improvement in CVH, particularly among younger adults, may reflect increased health awareness and greater engagement in preventive behaviors [36]. Surveys conducted during and after the COVID-19 pandemic documented changes in health-related habits, including increased physical activity, improved sleep, and reduced smoking in some subgroups [37,38]. However, these changes were not uniform. While blood pressure control and physical activity improved modestly, dietary quality and glycemic control deteriorated [36,39]. These trends likely reflect the unequal impact of the pandemic, i.e., some individuals adopted healthier routines, while others experienced reduced access to care or increased psychosocial stress. Older adults and socioeconomically disadvantaged groups appear to have been particularly vulnerable to these disruptions [35,40].

When examined by individual component, trends in CVH metrics revealed a heterogeneous pattern. Smoking prevalence declined modestly, aligning with global efforts to reduce tobacco use [41]. Conversely, dietary quality deteriorated, as indicated by a reduction in fruit and vegetable consumption. Physical activity showed slight improvements, more pronounced among younger individuals and women. BMI trends suggested a modest improvement, particularly in younger adults, although excess weight remains widespread. Blood pressure and total cholesterol metrics improved steadily, likely reflecting enhanced detection, treatment adherence, and broader therapeutic coverage [42,43]. In contrast, glycemic values worsened across calendar years, especially among older men, consistent with international evidence of rising HbA1c levels and reduced achievement of glycemic targets [42,43].

Together, these findings highlight both encouraging progress and ongoing challenges in the promotion of CVH in Italy. While overall CVH scores have improved over the past decade, particularly among younger adults, significant disparities by age and sex persist. These differences likely reflect not only current risk factor profiles but also the long-term accumulation of suboptimal behaviors and exposures, especially during midlife. CVD typically develops over decades, and low CVH observed in older adults may originate from insufficient prevention and risk factor control earlier in life [3,4]. Therefore, prevention strategies should adopt a lifecourse perspective. Targeted educational interventions in midlife, especially among men, combined with community-based programs accessible across age groups, may contribute to reducing long-term inequalities and promoting more equitable cardiovascular outcomes. Building on the infrastructure of PASSI and PASSI d’Argento, the inclusion of standardized composite CVH scores in monitoring systems could offer a more holistic view of population health. At the policy level, the growing international emphasis on physical activity, reflected in the WHO Global Action Plan and its adoption within European Union frameworks, has prompted the development of national health-enhancing physical activity policies and surveillance systems across Europe, including Italy. These initiatives have supported cross-sectoral efforts in education, transport, urban planning, and workplace settings, contributing to more coordinated responses to physical inactivity [44]. Nonetheless, several gaps remain, particularly in the monitoring of upstream determinants and in the harmonization of surveillance indicators across regions and systems [45]. To reduce barriers to physical activity and promote healthier lifestyles across the life course, further investments are needed in structural and organizational facilitators [46]. Within this broader context, individual-level engagement remains a key pillar. The LS7 and LE8 frameworks offer a practical structure for promoting self-awareness and encouraging behavior change through clearly defined, measurable health targets [3,4]. Evidence suggests that individuals who are informed about their CVH status and can monitor their own progress are more likely to adopt and sustain healthy behaviors over time [47]. Crucially, the burden of CVD is not limited to individuals identified as high risk. As shown by Leong et al. [48], a substantial proportion of cardiovascular events occur among those classified as low or intermediate risk using conventional algorithms. This supports the adoption of a dual prevention model, combining targeted interventions for high-risk individuals with broader, scalable strategies aimed at shifting the overall distribution of risk factors in the population. Community-based interventions that leverage local resources and address social determinants of health are also essential [46]. Successful implementation will require interdisciplinary collaboration, digital and mobile health solutions to extend reach, and sustained community engagement [49]. Equity must remain central, with locally tailored programs to reduce disparities in care and outcomes [6]. Looking ahead, the Lookup 8+ project provides a scalable framework for real-world cardiovascular health surveillance. Continuous monitoring of CVH metrics beyond clinical settings can complement existing registries and help identify emerging trends in population risk. Incorporating such indicators into prevention and health policy frameworks may foster more proactive and equitable cardiovascular care. Moreover, the findings highlight the importance of coupling individual-level behavior change with structural and policy actions addressing upstream determinants, such as health literacy, urban design, and access to healthy environments. The evolving trends described in this report may ultimately guide tailored prevention programs and inform long-term strategies to reduce the cardiovascular disease burden in aging societies.

### Study Limitations

Despite reporting novel and policy-relevant findings on cardiovascular health trends in a large, community-based Italian cohort, this study has several limitations that warrant consideration. First, its cross-sectional design precludes causal inference and does not allow for longitudinal tracking of individual trajectories in CVH. While trends were evaluated across calendar years, they reflect population-level changes rather than within-subject evolution. Second, although recruitment was conducted across diverse Italian regions and included adults aged 18 years and older, the sample is not nationally representative. Participants were enrolled through voluntary walk-in campaigns, potentially introducing selection bias toward more health-conscious individuals or those with better access to screening opportunities. Third, data on smoking, diet, physical activity, and medication use were self-reported, raising the possibility of recall or social desirability bias. The brief nature of assessments, particularly for diet, may have limited accuracy. Fourth, the operationalization of CVH metrics, e.g., physical activity and healthy diet, was pragmatically adapted to facilitate community-based assessments and may not fully align with current international recommendations. Physical activity was classified based on self-reported frequency and duration (≥30 min, ≥2 times/week), which served as a simplified proxy measure of habitual activity levels, in line with feasible approaches for population-based assessment of physical activity [50]. Fifth, no information was available on disease conditions. However, given the public and non-clinical nature of recruitment settings, participants were presumed to be generally healthy at the time of enrollment. In particular, data on prior cardiovascular conditions or procedures (including coronary revascularization or stenting) were not collected, as the Lookup 8+ project was conceived for community-based screening rather than clinical profiling; this pragmatic design allowed standardized, rapid, and non-invasive assessments across large populations, but inevitably limited the availability of detailed medical history. Sixth, sleep health was not included among the analyzed CVH metrics, as this domain was incorporated into the Lookup project only after 2019 and formally introduced in the LE8 framework in 2022. Nevertheless, recent analyses from our group have examined sleep health within an extended construct inspired by the LE8 framework [51]. Incorporating this domain in future population-based surveillance efforts may offer a more comprehensive view of cardiovascular health trajectories and behavioral determinants. Seventh, cholesterol and glucose were measured using standardized point-of-care capillary devices rather than fasting venous samples, which may limit comparability with standard laboratory data. In addition, only total cholesterol was available, with no data on HDL or LDL fractions, which precluded assessment of individual lipid components and limited the ability to fully characterize participants’ lipid profiles. Finally, although models were adjusted for age, sex, and year of assessment, unmeasured confounders, such as socioeconomic status, education, and healthcare access, may have influenced observed patterns. Residential environment (urban or rural), educational attainment, and income were excluded from data collection to mitigate the potential for discomfort or embarrassment among participants with limited formal education or financial resources. This approach was adopted to foster a supportive environment and promote positive engagement throughout the assessment process [52]. Future studies with broader social and clinical data are needed to validate these findings.

## 5. Conclusions

This ten-year report from the Lookup study offers a comprehensive overview of cardiovascular health trends in the Italian adult population. The observed improvements in overall CVH, particularly among younger individuals, are encouraging; however, substantial disparities by age and sex remain. These findings underscore the need for integrated, life-course approaches to prevention, combining individual-level engagement with structural and policy-level strategies. Inftegrating CVH monitoring and equitable prevention will be essential to advance cardiovascular health in aging societies.

## Figures and Tables

**Table 1:** Population characteristics by age group.

	18–39 Years (*N* = 2599)	40–64 Years (*N* = 10,242)	≥65 Years (*N* = 5650)	Total Sample (*N* = 18,491)
Age, years	30.4 ± 6.1	53.5 ± 6.5	72.7 ± 5.9	56.1 ± 14.8
Sex, female	1453 (55.9%)	5750 (56.1%)	3013 (53.3%)	10,216 (55.2%)
BMI, kg/m^2^	23.3 ± 3.9	25.0 ± 4.1	26.1 ± 4.0	25.1 ± 4.1
Systolic blood pressure, mmHg	116.9 ± 13.4	123.7 ± 16.0	131.0 ± 16.6	125.0 ± 16.5
Diastolic blood pressure, mmHg	72.3 ± 9.2	76.4 ± 10.0	76.2 ± 9.7	75.8 ± 9.9
On antihypertensive therapy	45 (1.7%)	1915 (18.7%)	2958 (52.4%)	4918 (26.6%)
Total cholesterol, mg/dL	189.7 ± 34.3	202.9 ± 37.0	196.2 ± 36.8	199.0 ± 36.9
On lipid-lowering therapy	38 (1.5%)	1337 (13.1%)	1980 (35.0%)	3355 (18.1%)
Blood glucose, mg/dL	98.5 ± 18.3	103.1 ± 21.9	110.4 ± 26.8	104.7 ± 23.4
On glucose-lowering therapy	24 (0.9%)	269 (2.6%)	521 (9.2%)	814 (4.4%)
CVH score	4.9 ± 1.3	4.2 ± 1.4	3.8 ± 1.3	4.2 ± 1.4
Low CVH score (0–2)	113 (4.3%)	1154 (11.3%)	851 (15.1%)	2118 (11.5%)
Intermediate CVH score (3–4)	824 (31.7%)	4579 (44.7%)	3129 (55.4%)	8532 (46.1%)
High CVH score (5–7)	1662 (63.9%)	4509 (44.0%)	1670 (29.6%)	7841 (42.4%)

Continuous variables are presented as mean ± standard deviation; categorical variables are reported as absolute numbers with percentages. Abbreviations: BMI, body mass index; CVH, cardiovascular health.

**Table 2:** Multivariable logistic regression models assessing factors associated with ideal cardiovascular health (cardiovascular health score ≥ 5).

	Model 1 OR (95% CI)	*p*	Model 2 OR (95% CI)	*p*	Model 3 OR (95% CI)	*p*	Model 4 OR (95% CI)	*p*
Age	0.97 (0.96–0.97)	<0.001	0.97 (0.97–0.97)	<0.001	0.97 (0.96–0.97)	<0.001	0.97 (0.97–0.97)	<0.001
Sex, female	1.64 (1.55–1.75)	<0.001	2.25 (1.76–2.89)	<0.001	1.64 (1.54–1.81)	<0.001	2.28 (1.77–2.94)	<0.001
Post-COVID-19	1.32 (1.24–1.40)	<0.001	1.64 (1.54–1.75)	<0.001	1.34 (1.23–1.48)	<0.001	1.66 (1.53–1.80)	<0.001
Age × post-COVID-19 interaction	–	–	0.99 (0.99–1.00)	<0.001	–	–	0.99 (0.99–1.00)	<0.001
Female sex × post-COVID-19 interaction					0.97 (0.86–1.10)	0.600	0.98 (0.86–1.11)	0.700

Table shows odds ratios (ORs) and 95% confidence intervals (CIs) for each covariate. Model 1 includes age, sex, and the post-COVID period (defined as year ≥ 2020). Model 2 introduces an interaction term between age and the post-COVID period. Model 3 includes an interaction between sex and the post-COVID period. Model 4 incorporates both interaction terms. Ideal cardiovascular health is defined as a cardiovascular health score ≥ 5, with higher scores indicating more favorable cardiovascular profiles.

## Data Availability

The data that support the findings of this study are available on request from the corresponding author. The data are not publicly available due to their containing information that could compromise the privacy of the research participants.

## Supplementary Materials

The following supporting information can be downloaded at https://www.mdpi.com/article/10.3390/medsci13040251/s1, Supplementary Table S1. Prevalence of the seven cardiovascular health metrics according to age-groups. Supplementary Table S2. Linear regression model assessing the association between year of assessment and Cardiovascular Health Score, adjusted for age group and sex. CI: confidence interval. Supplementary Table S3. Logistic regression models evaluating temporal and demographic correlates of individual non-ideal cardiovascular health metrics. Supplementary Figure S1. Temporal trends in the age-standardized. Supplementary Figure S2. Temporal trends in the age-standardized prevalence of unhealthy diet from 2015 to 2024 stratified by age group. The red dashed line indicates the start of the COVID-19 pandemic. Supplementary Figure S3. Temporal trends in the age-standardized prevalence of low physical activity from 2015 to 2024 stratified by age group. The red dashed line indicates the start of the COVID-19 pandemic. Supplementary Figure S4. Temporal trends in the age-standardized prevalence of non-ideal cholesterol from 2015 to 2024 stratified by age group. The red dashed line indicates the start of the COVID-19 pandemic. Supplementary Figure S5. Temporal trends in the age-standardized prevalence of non-ideal glycemic control from 2015 to 2024 stratified by age group. The red dashed line indicates the start of the COVID-19 pandemic. Supplementary Figure S6. Temporal trends in the age-standardized prevalence of non-ideal blood pressure from 2015 to 2024 stratified by age group. The red dashed line indicates the start of the COVID-19 pandemic. Supplementary Figure S7. Temporal trends in the age-standardized prevalence of non-ideal body mass index from 2015 to 2024 stratified by age group. The red dashed line indicates the start of the COVID-19 pandemic. Supplementary Figure S8. Temporal trends in the sex-standardized prevalence of active smoking from 2015 to 2024 stratified by sex. The red dashed line indicates the start of the COVID-19 pandemic. Supplementary Figure S9. Temporal trends in the sex-standardized prevalence of unhealthy diet from 2015 to 2024 stratified by age group. The red dashed line indicates the start of the COVID-19 pandemic. Supplementary Figure S10. Temporal trends in the sex-standardized prevalence of low physical activity from 2015 to 2024 stratified by age group. The red dashed line indicates the start of the COVID-19 pandemic. Supplementary Figure S11. Temporal trends in the sex-standardized prevalence of non-ideal cholesterol from 2015 to 2024 stratified by age group. The red dashed line indicates the start of the COVID-19 pandemic. Supplementary Figure S12. Temporal trends in the sex-standardized prevalence of non-ideal glycemic control from 2015 to 2024 stratified by age group. The red dashed line indicates the start of the COVID-19 pandemic. Supplementary Figure S13. Temporal trends in the sex-standardized prevalence of non-ideal blood pressure from 2015 to 2024 stratified by age group. The red dashed line indicates the start of the COVID-19 pandemic. Supplementary Figure S14. Temporal trends in the sex-standardized prevalence of non-ideal body mass index from 2015 to 2024 stratified by age group. The red dashed line indicates the start of the COVID-19 pandemic.
