# Supplementary material for: Temporal Trends in Cardiovascular Health Metrics in Italy, 2015–2024: A Ten-Year Report from the Longevity Check-Up (Lookup) 8+ Study
Source: Med Sci (Basel). 2025 Oct 30;13(4):251. doi: 10.3390/medsci13040251 (PMC12641886; doi:10.3390/medsci13040251)
Supplement: Supplementary file 1 [file medsci-13-00251-s001.zip › medsci-3928724-supplementary.pdf]

# Temporal Trends in Cardiovascular Health Metrics in Italy, 2015–2024: A Ten-Year Report from the Longevity Check-Up (Lookup) 8+ Study

**Stefano Cacciatore, Elena Levati, Riccardo Calvani, Matteo Tosato, Francesca Ciciarello, Vincenzo Galluzzo, Sara Salini, Andrea Russo, Emanuele Marzetti, Francesco Landi**

**Supplementary Table S1.** Prevalence of the seven cardiovascular health metrics according to age-groups.

| | **18–39 years (N= 2,599)** | **40–64 years (N= 10,242)** | **≥ 65 years (N= 5,650)** | **Total sample (N=18,491)** |
|---|---|---|---|---|
| **Smoking status** | | | | |
| Never smoker | 1,558 (59.9%) | 5916 (57.8%) | 3154 (55.8%) | 10628 (57.5%) |
| Former smoker | 443 (17.0%) | 2544 (24.8%) | 1908 (33.8%) | 4895 (26.5%) |
| Current smoker | 596 (22.9%) | 1773 (17.3%) | 575 (10.2%) | 2944 (15.9%) |
| **BMI** | | | | |
| < 25 kg/m² | 1891 (72.8%) | 5689 (55.5%) | 2389 (42.3%) | 9969 (53.9%) |
| 25.0-29.9 kg/m² | 563 (21.7%) | 3434 (33.5%) | 2376 (42.1%) | 6373 (34.5%) |
| ≥ 30 kg/m² | 145 (5.6%) | 1119 (10.9%) | 885 (15.7%) | 2149 (11.6%) |
| **Healthy diet** | | | | |
| Yes | 1461 (56.2%) | 6342 (61.9%) | 4233 (74.9%) | 12036 (65.1%) |
| No | 906 (34.9%) | 4275 (41.7%) | 2484 (44.0%) | 7665 (41.5%) |
| **Regular physical activity** | | | | |
| Yes | 1693 (65.1%) | 5967 (58.3%) | 3166 (56.0%) | 10826 (58.5%) |
| No | 906 (34.9%) | 4275 (41.7%) | 2484 (44.0%) | 7665 (41.5%) |
| **Blood pressure** | | | | |
| ≤120/80 mmHg (untreated) | 1738 (66.9%) | 4461 (43.6%) | 986 (17.5%) | 7185 (38.9%) |
| 121–139/81–89 mmHg (or treated to target) | 608 (23.4%) | 3272 (31.9%) | 2558 (45.3%) | 6438 (34.8%) |
| ≥140/≥90 mmHg | 253 (9.7%) | 2509 (24.5%) | 2106 (37.3%) | 4868 (26.3%) |
| **Total cholesterol** | | | | |
| <200 mg/dL | 1640 (63.1%) | 4221 (41.2%) | 1822 (32.2%) | 7683 (41.6%) |
| 200–239 mg/dL or treated to target | 758 (29.2%) | 4388 (42.8%) | 3176 (56.2%) | 8322 (45.0%) |
| >240 mg/dL | 201 (7.7%) | 1633 (15.9%) | 652 (11.5%) | 2486 (13.4%) |
| **Glycemic control** | | | | |

| | | | | |
|---|---|---|---|---|
| Normal | 2316 (89.1%) | 8276 (80.8%) | 3826 (67.7%) | 14418 (78.0%) |
| Suboptimal | 283 (10.9%) | 1966 (19.2%) | 1824 (32.3%) | 4073 (22.0%) |

Continuous variables are presented as mean ± standard deviation; categorical variables are reported as absolute numbers with corresponding percentages. Abbreviation: BMI, body mass index.

**Supplementary Table S2.** Linear regression model assessing the association between year of assessment and Cardiovascular Health Score, adjusted for age group and sex. CI: confidence interval.

| Characteristic | β (95% CI) | p-value |
|---|---|---|
| Year of assessment | | |
| 2015 (reference) | — | — |
| 2016 | –0.34 (–0.43 to –0.26) | <0.001 |
| 2017 | –0.41 (–0.48 to –0.34) | <0.001 |
| 2018 | –0.15 (–0.22 to –0.08) | <0.001 |
| 2019 | 0.16 (0.05 to 0.28) | 0.005 |
| 2020 | –0.09 (–0.24 to 0.07) | 0.300 |
| 2021 | –0.13 (–0.22 to –0.03) | 0.009 |
| 2022 | –0.11 (–0.19 to –0.02) | 0.011 |
| 2023 | 0.00 (–0.07 to 0.08) | 0.999 |
| 2024 | 0.01 (–0.06 to 0.08) | 0.700 |
| Sex | | |
| Male (reference) | — | — |
| Female | 0.40 (0.36 to 0.43) | <0.001 |
| Age group | | |
| 18–39 years (reference) | — | — |
| 40–64 years | –0.65 (–0.71 to –0.59) | <0.001 |
| ≥65 years | –1.10 (–1.10 to –0.99) | <0.001 |

**Supplementary Table S3.** Logistic regression models evaluating temporal and demographic correlates of individual non-ideal cardiovascular health metrics.

| | Age | Sex, female | Post-COVID-19 |
|---|---|---|---|
| **Active smoking OR (95% CI), p** | 0.98 (0.98–0.98), <0.001 | 0.91 (0.84–0.98), 0.018 | 0.94 (0.86–1.01), 0.100 |
| **Unhealthy diet OR (95% CI), p** | 0.98 (0.98–0.98), <0.001 | 0.60 (0.54–0.64), <0.001 | 1.57 (1.48–1.67), <0.001 |
| **Low physical activity OR (95% CI), p** | 1.01 (1.01–1.01), <0.001 | 1.36 (1.28–1.45), <0.001 | 0.72 (0.68–0.76), <0.001 |
| **Non-ideal cholesterol OR (95% CI), p** | 1.03 (1.03–1.03), <0.001 | 1.52 (1.43–1.62), <0.001 | 0.58 (0.55–0.62), <0.001 |
| **Non-ideal glycemic control OR (95% CI), p** | 1.03 (1.03–1.04), <0.001 | 0.77 (0.71–0.82), <0.001 | 2.07 (1.92–2.22), <0.001 |
| **Non-ideal blood pressure OR (95% CI), p** | 1.06 (1.06–1.06), <0.001 | 0.40 (0.37–0.43), <0.001 | 0.69 (0.65–0.74), <0.001 |
| **Non-ideal body mass index OR (95% CI), p** | 1.03 (1.03–1.03), <0.001 | 0.43 (0.40–0.46), <0.001 | 0.64 (0.60–0.68), <0.001 |

For each of the seven health metrics, logistic regression models were used to estimate the association of age, sex, and the post-COVID period (year ≥2020) with the likelihood of exhibiting a non-ideal status. Results are expressed as odds ratios (ORs) with 95% confidence intervals (CIs) and p values.

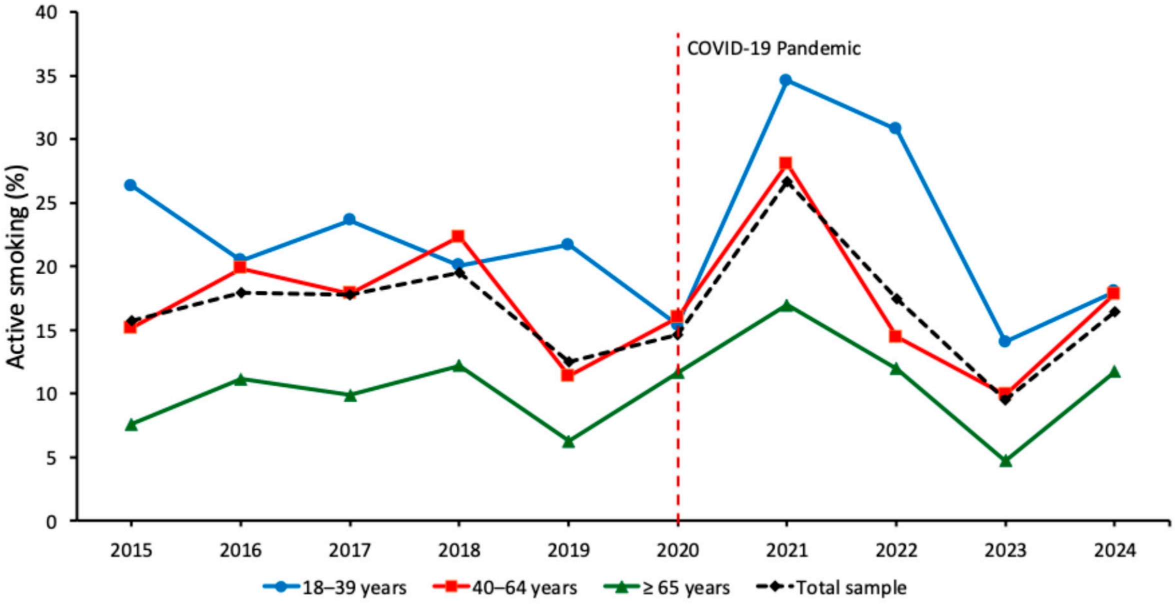


**Supplementary Figure S1.** Temporal trends in the age-standardized prevalence of active smoking from 2015 to 2024 stratified by age group. The red dashed line indicates the start of the COVID-19 pandemic.

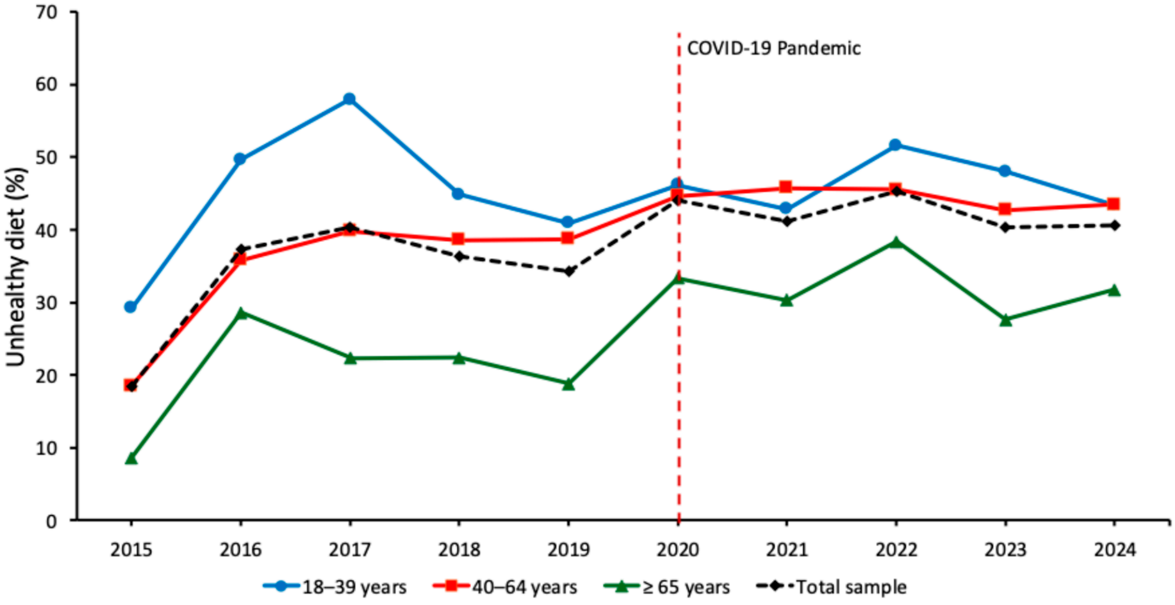


**Supplementary Figure S2.** Temporal trends in the age-standardized prevalence of unhealthy diet from 2015 to 2024 stratified by age group. The red dashed line indicates the start of the COVID-19 pandemic.

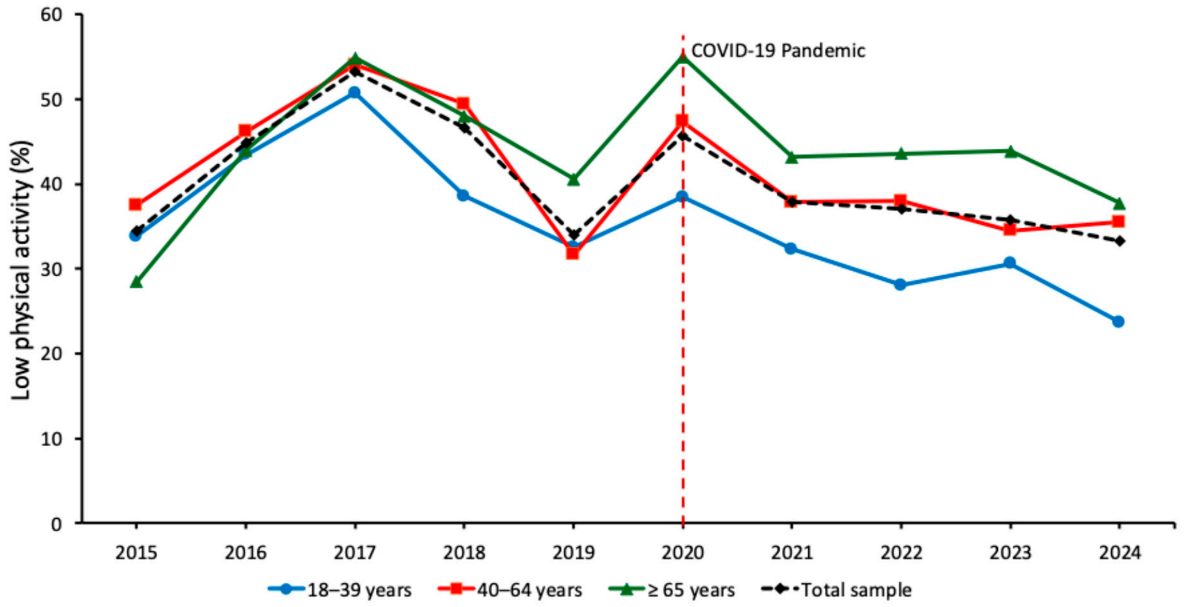


**Supplementary Figure S3.** Temporal trends in the age-standardized prevalence of low physical activity from 2015 to 2024 stratified by age group. The red dashed line indicates the start of the COVID-19 pandemic.

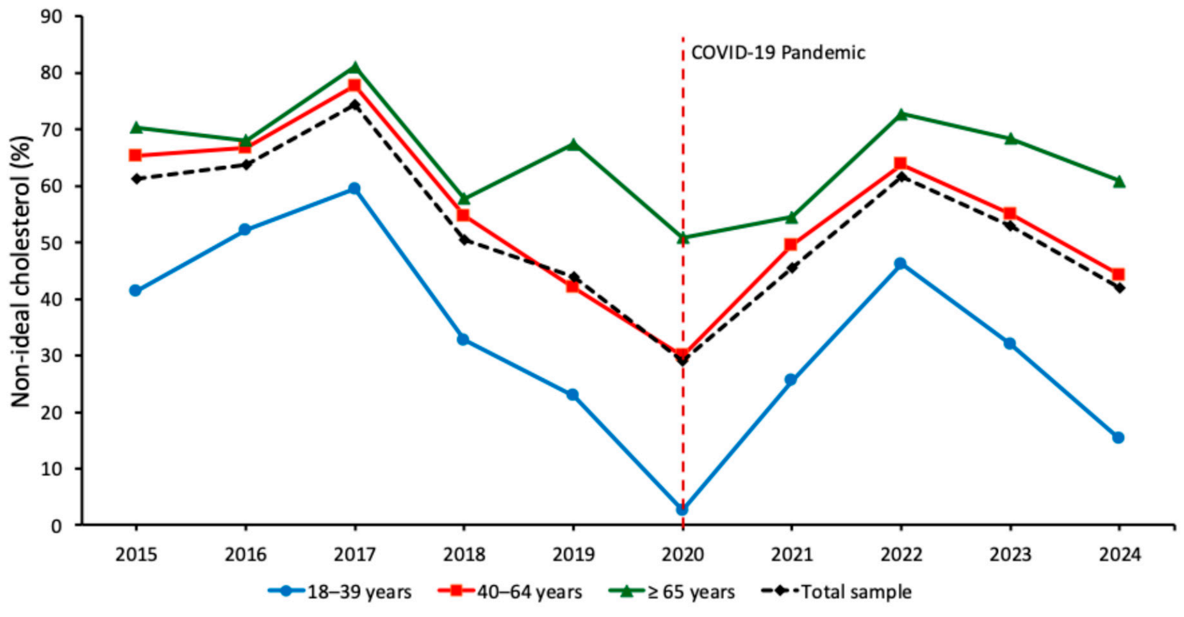


**Supplementary Figure S4.** Temporal trends in the age-standardized prevalence of non-ideal cholesterol from 2015 to 2024 stratified by age group. The red dashed line indicates the start of the COVID-19 pandemic.

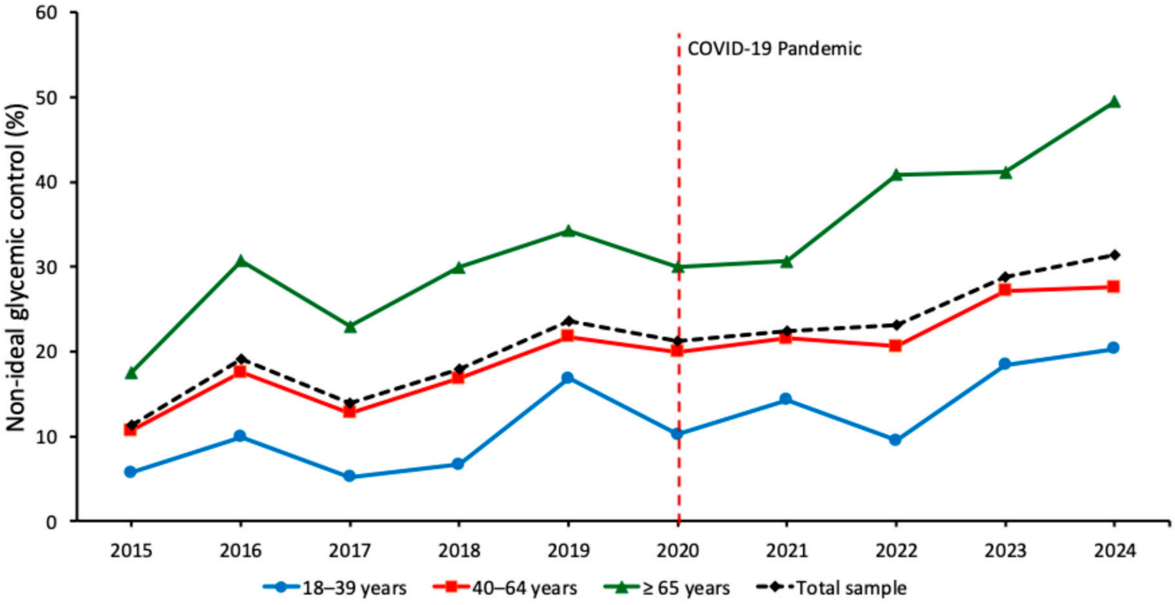


**Supplementary Figure S5.** Temporal trends in the age-standardized prevalence of non-ideal glycemic control from 2015 to 2024 stratified by age group. The red dashed line indicates the start of the COVID-19 pandemic.

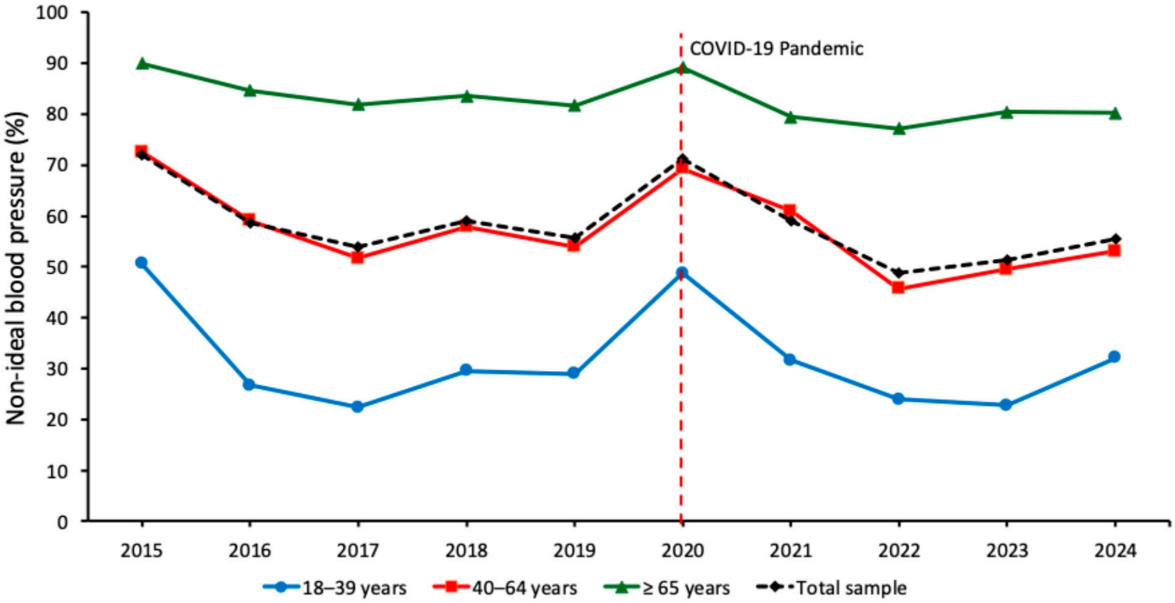


**Supplementary Figure S6.** Temporal trends in the age-standardized prevalence of non-ideal blood pressure from 2015 to 2024 stratified by age group. The red dashed line indicates the start of the COVID-19 pandemic.

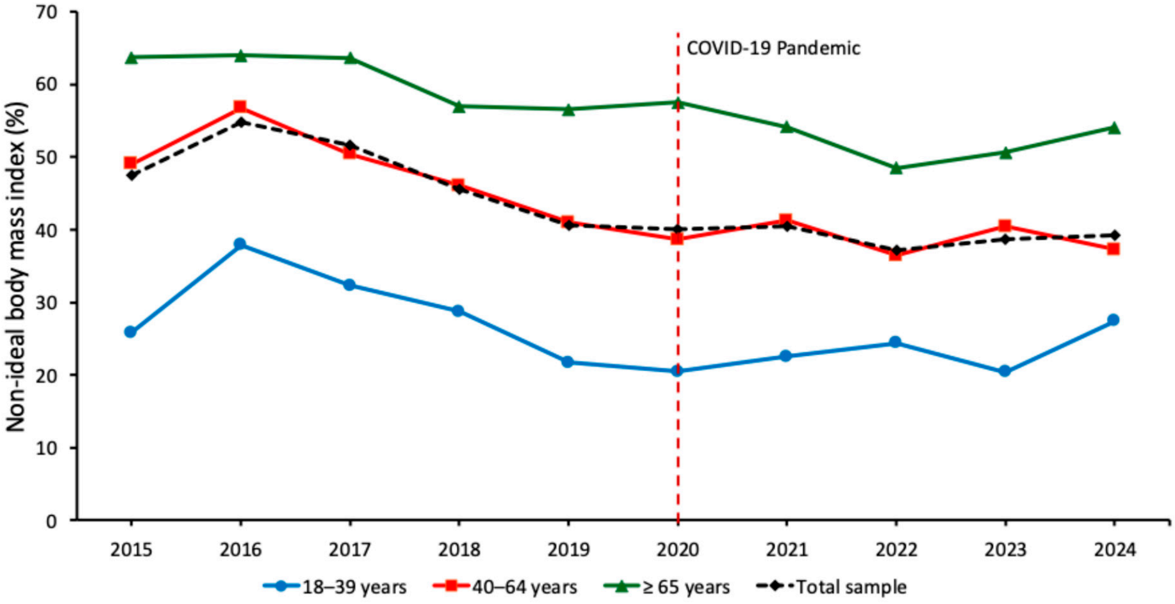


**Supplementary Figure S7.** Temporal trends in the age-standardized prevalence of non-ideal body mass index from 2015 to 2024 stratified by age group. The red dashed line indicates the start of the COVID-19 pandemic.

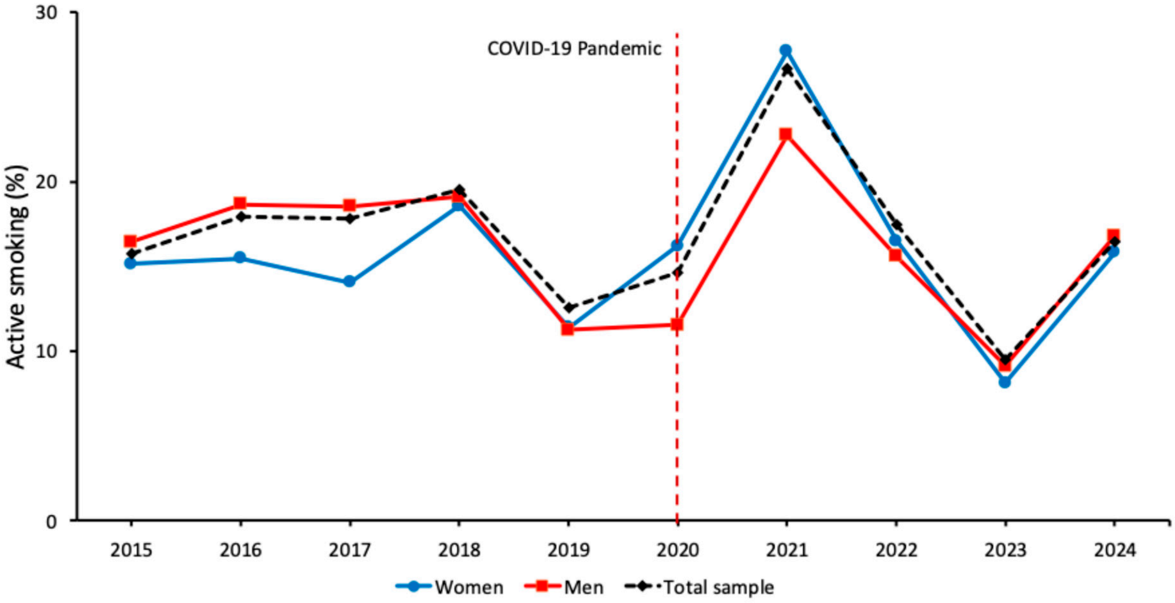


**Supplementary Figure S8.** Temporal trends in the sex-standardized prevalence of active smoking from 2015 to 2024 stratified by sex. The red dashed line indicates the start of the COVID-19 pandemic.

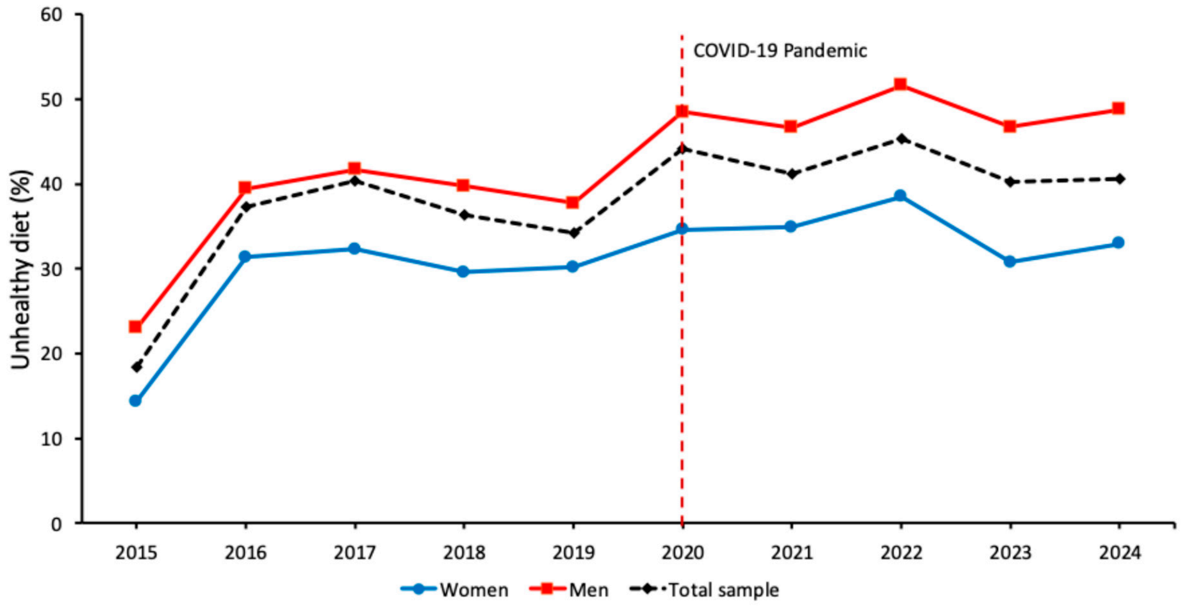


**Supplementary Figure 9.** Temporal trends in the sex-standardized prevalence of unhealthy diet from 2015 to 2024 stratified by age group. The red dashed line indicates the start of the COVID-19 pandemic.

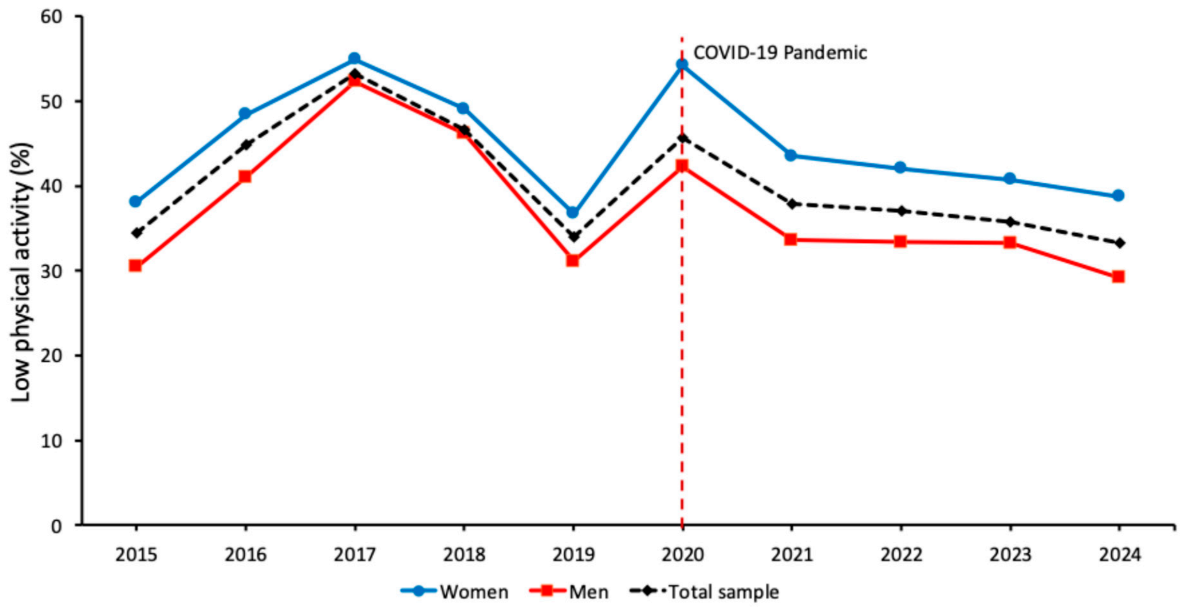


**Supplementary Figure S10.** Temporal trends in the sex-standardized prevalence of low physical activity from 2015 to 2024 stratified by age group. The red dashed line indicates the start of the COVID-19 pandemic.

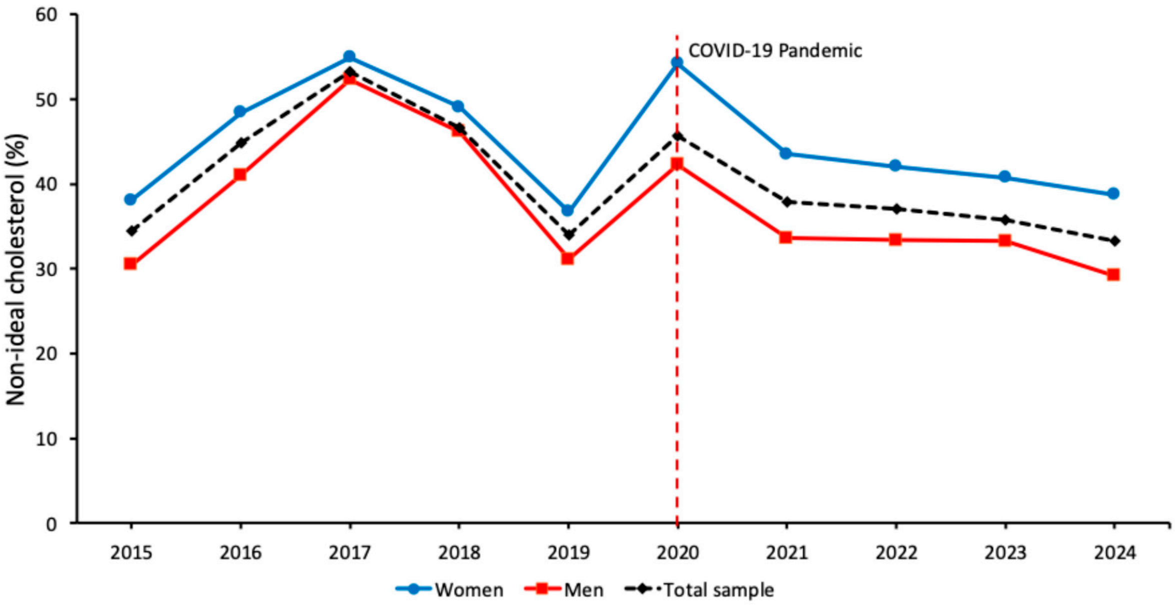


**Supplementary Figure S11.** Temporal trends in the sex-standardized prevalence of non-ideal cholesterol from 2015 to 2024 stratified by age group. The red dashed line indicates the start of the COVID-19 pandemic.

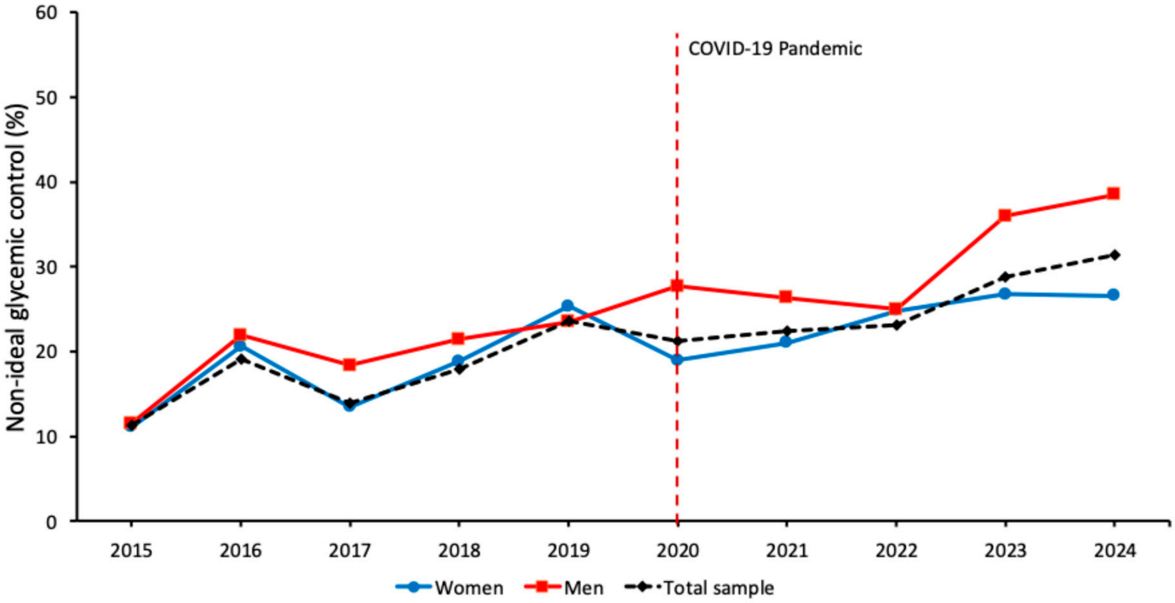


**Supplementary Figure S12.** Temporal trends in the sex-standardized prevalence of non-ideal glycemic control from 2015 to 2024 stratified by age group. The red dashed line indicates the start of the COVID-19 pandemic.

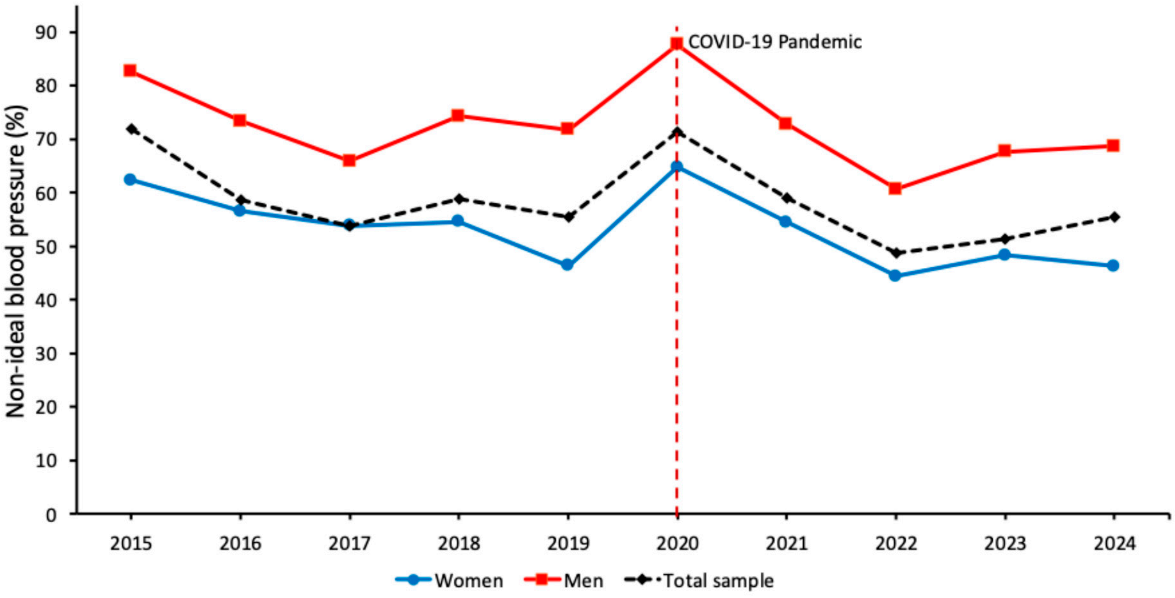


**Supplementary Figure S13.** Temporal trends in the sex-standardized prevalence of non-ideal blood pressure from 2015 to 2024 stratified by age group. The red dashed line indicates the start of the COVID-19 pandemic.

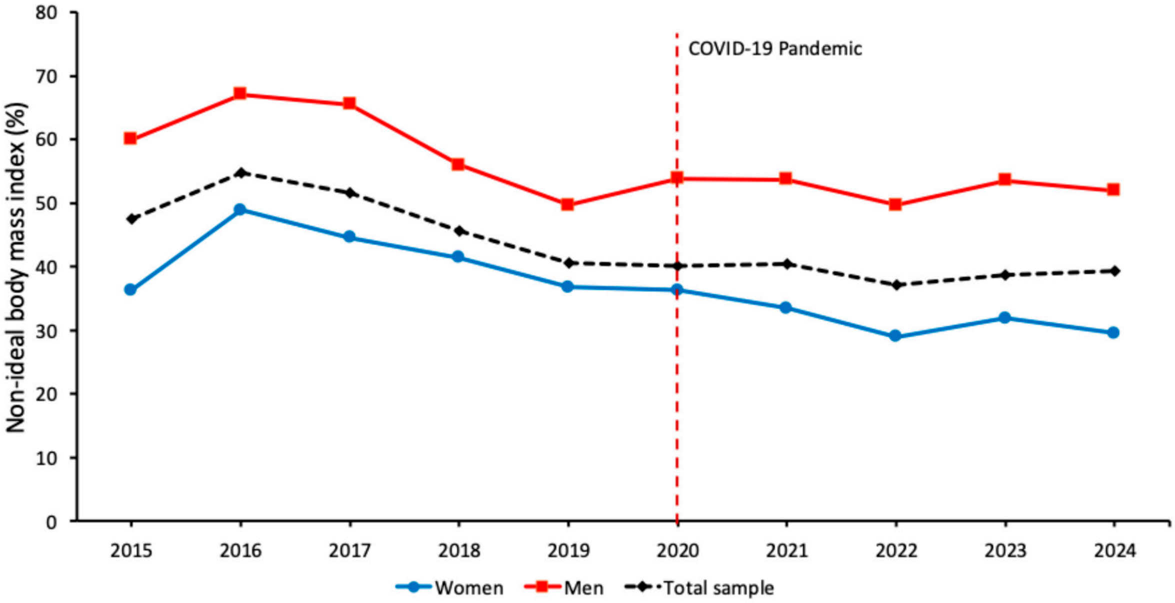


**Supplementary Figure S14.** Temporal trends in the sex-standardized prevalence of non-ideal body mass index from 2015 to 2024 stratified by age group. The red dashed line indicates the start of the COVID-19 pandemic.
